# Eye care in South Asia, 1988–2018: developments, achievements and future challenges

**Published:** 2018-02-08

**Authors:** Gudlavalleti VS Murthy

**Affiliations:** 1 Director: South Asia Centre for Disability Inclusive Development, Indian Institute of Public Health and Professor: International Centre for Eye Health, London School of Hygiene & Tropical Medicine, London, UK.

**Despite South Asia's many challenges, including a rapid increase in non-communicable eye diseases such as glaucoma, diabetic retinopathy and retinopathy of prematurity, the many and varied successes of the last thirty years are a cause for celebration.**

**Figure:**
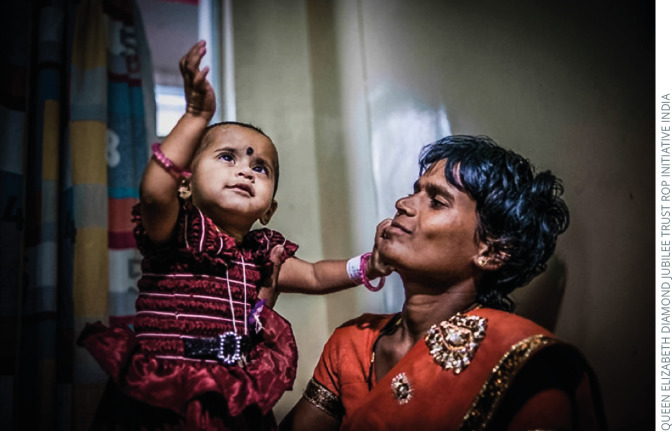
Health must be at the centre of political debate in South Asia. INDIA

South Asia is a unique geopolitical region which covers 3.4% of the world's surface area and supports 25% of the world's population (1.749 billion). It hosts the second (India), sixth (Pakistan) and the eighth (Bangladesh) most populous countries in the world. The South Asia Association for Regional Cooperation (SAARC) was established in 1985 and currently has eight Member States. SAARC accounts for a tenth of the global economy and member countries span two World Health Organization (WHO) regions: Eastern Mediterranean (Afghanistan, Pakistan) and South East Asia (Bangladesh, Bhutan, India, Maldives, Nepal and Sri Lanka). Over the past three decades, South Asia has faced and withstood many human and natural disasters, including a tsunami, devastating cyclones, annual floods, earthquakes, landslides, riots, civil disturbances, wars and terrorist strikes.

## Developments in eye health: 1988–2018

South Asia has witnessed a dramatic demographic transformation over the last three decades, with the population increasing by 162%. Life expectancy at birth across South Asia has increased by 11 years, from 57 years in 1988 to 68 years in 2015, with the increase ranging from 6 years in Pakistan to 19 years in Bhutan. Infant and under-five mortality rates have decreased in all countries in South Asia from 1988 to 2016 and the population aged 50 years and older has increased significantly. This demographic transition has increased the ‘at-risk’ population for visual impairment and blindness as all the commonest causes are age-related. At the same time, with decreasing under-five mortality rates, the prevalence and causes of childhood blindness have decreased, particularly the nutritional and infective causes.

There are two main challenges facing South Asia. Visual impairment and blindness due to infectious conditions (such as leprosy and trachoma) still exist, and non-communicable blinding conditions such as cataract, glaucoma, diabetic retinopathy (DR) and retinopathy of prematurity (ROP) are increasing rapidly in magnitude. As a result of these challenges, and the failure of the health systems in the region to provide adequate services (due to logistical issues), conditions such as uncorrected refractive error still require urgent attention in South Asia – despite the fact that the technical know-how exists.

### Eye health and prevention of blindness

South Asia is home to four WHO Collaborative Centres for Prevention of Blindness (one in Pakistan and three in India). Most countries in South Asia have a mixed health care system. There is significant participation from the private sector, including non-governmental organisations (NGOs), especially at the secondary and tertiary levels. At the primary level, the government is the principal service provider. In Sri Lanka, Bhutan and Maldives, eye care is predominantly managed by the government, whereas in Nepal and Bangladesh the NGO sector is the predominant service provider. On average, individuals have to pay 61% of the cost of health care out of their own pockets. It is lowest in the Maldives (18%) and highest Bangladesh (67%).

South Asia, along with neighbouring countries in South East Asia, has been the cradle of innovation in eye care since time immemorial, starting with outreach eye camps for cataract surgery and pilot programmes for trachoma control. India was the first country to establish a national programme for control of blindness (in 1976). It was declared a national priority by the then Prime Minister of India and later many countries in the region adopted it as an effective approach to addressing blindness.

The year 1988 began with the reaffirmation of the Alma-Ata ‘Health for all’ declaration in Riga, Latvia – ten years after it first identified primary health care as the key to the attainment of the goal of Health for All. Along with the emerging epidemic of HIV/AIDS, this led to increased emphasis on primary health care worldwide. Countries in South Asia initiated schemes such as Lady Health Workers in Pakistan and Community Health Workers (CHWs) and Accredited Social Health Activists in India. In countries where primary health care development has been strong and a functional system exists, eye care programmes have been more successful.

Many international non-governmental organisations support eye care work in South Asia. As a result, infrastructure has improved and human resources for eye health have been augmented.^1^

### Blindness and visual impairment

The Global Burden of Disease study observed that South Asia is the region that is home to the greatest number of people who are blind (11.7 million), which accounts for 32.5% of the people who are blind worldwide. The prevalence of blindness in South Asia was 0.7%. Similarly, 61.2 million people in South Asia have moderate and severe visual impairment, which accounts for 28.2% of the global magnitude.^2^

Despite the challenges, much progress has been made in reducing avoidable blindness. From 1990 to 2015, the age-standardised prevalence rate of blindness is estimated to have reduced from 0.8% to 0.5% (all ages), and from 5.2% to 3.5% among people aged 50 years and older. Population-based surveys repeated in the same geographical areas in India, 10 years apart, have demonstrated this reduction very effectively.^3^ Eye care indicators in South Asia reveal a significant decrease in the prevalence of blindness and a continuous increase of cataract surgical rates (CSR) and cataract surgical coverage (CSC).

### VISION 2020

The regional launches of VISION 2020 in both the Eastern Mediterranean and South-East Asia regions took place in September 1999.^4^ This added pace and energy to the eye care efforts in the regions and have resulted in programme advocacy with the political and administrative leadership in individual countries. A VISION 2020 action plan has been operational in all countries in South Asia since 2010.

## Achievements in eye care (1988–2016)

South Asia has contributed success stories to augment the global initiative for the elimination of avoidable blindness over the past three decades. The following examples highlight this contribution.

**Figure:**
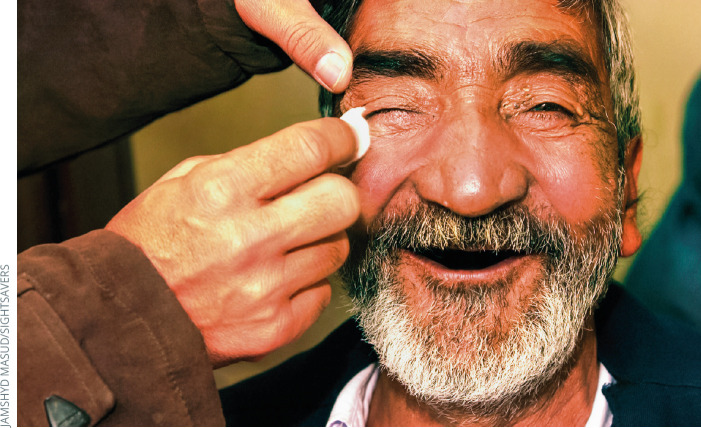
Cataract surgery has restored this man's vision. PAKISTAN

### Evidence for planning

1. Using evidence to plan eye care programmes has been the hallmark of the national programme for the control of blindness in India. Surveys have been conducted at periodic intervals and have guided the evolution of eye care programmes, especially over the last three decades. For example, the National Trachoma Control Program was later transformed into a more comprehensive National Program for Control of Blindness in order to address national eye health priorities.
2. Development and validation of the rapid assessment tools for blindness and cataract surgical services took place in India during the period 1992–1996. This was the precursor of the rapid assessment of avoidable blindness (RAAB) survey protocol (pp. 82–84).
3. Self-monitoring and institutional monitoring records for cataract surgery were first introduced in India in 1994.
4. Development and validation of the key informant method for childhood blindness was pioneered in Bangladesh in 1998.

### Improved quality and efficiency of eye care programmes

1. The high-volume cataract surgery protocol was developed and promoted by the Aravind Eye Care System in India in the 1980s and 1990s.
2. Universal use of intraocular lenses (IOL) in cataract surgery and the promotion of manual small-incision cataract surgery (MSICS) revolutionised cataract surgery. It led to excellent visual outcomes after surgery and cost reduction, which has improved access to cataract surgery for the poor. From a low of less than 5% in 1988, today more than 95% of the cataracts operated in South Asia receive an IOL.
3. The past three decades have seen a shift away from outreach surgical eye camps to a ‘source-and-serve’ approach: diagnosis takes place at outreach camps, and surgery is carried out at a base hospital. This approach improves outcomes and reduces the risk of complications, and has become the standard across most low- and middle income countries.
4. Integrating eye care into primary health care has been carried out successfully in India and Pakistan, where Accredited Social Health Activists and Lady Health Workers (respectively) have been trained as front-line health workers with the capacity to identify and refer eye problems as needed.
5. Development of a cost-effective school eye screening protocol began in 1992. School vision screening programmes were part of school health examinations, but vision was not emphasised. The reach of school eye screening was increased by developing a single optotype screening card (6/9) and training school teachers to do the initial screening. This reduced the workload of the few ophthalmic assistants/optometrists available and increased awareness among the school teachers and parents.

### Decentralised planning and service delivery

1. A comprehensive district eye care strategy – which invested in strengthening eye health infrastructure and human resource capacity for eye health at the district level-was the hallmark of the national plan in Pakistan in 1990. In 2008, a project was initiated to train 70,000 Lady Health Workers in primary eye care and provide them with appropriate skills and equipment.
2. In 1992, a pilot project was initiated in five districts in India to augment local planning capacity for eye care. District Blindness Control Societies were established and provided with partial financial and administrative autonomy to develop an annual programme implementation plan. This was then scaled up to the entire country from 1996 onwards and has found wide application as a planning template for eye care.

### National initiatives to control specific causes of avoidable blindness

1. A soft loan was obtained by the Indian government from the World Bank in 1994 to augment infrastructure and human resource capacity to tackle the magnitude of cataract and improve outcomes after surgery.
2. Special task forces – with strong political buy-in – have been set up in India to control visual impairment and blindness due to diabetic retinopathy and retinopathy of prematurity.

## Future challenges and way forward

### Financing

The foremost challenge is to ensure universal access to quality health care, including eye care, at an affordable cost for the country and with manageable out-of-pocket expenses for families. The inadequate financing of eye care services needs urgent attention. Models of affordable health insurance packages need to be formulated as the increasing proportion of middle-income and affluent sections of society can afford to share the costs of care.

### Non-communicable diseases

The epidemiologic transition is still continuing and the magnitude of non-communicable diseases is bound to increase further.^5^ The number of people with diabetes worldwide has been steadily increasing from an estimated 41.7 million in 2000 to 69.2 million in 2015 and is expected to increase to 124 million by 2040.^6^ The South Asia region is characterised by a high prevalence of type 2 diabetes, despite having a young population with relatively lower rates of obesity than that observed in high-income countries. This has been postulated to be the result of a ‘South Asian’ phenotype with higher waist circumference than in other countries, which has implications for diabetic retinopathy (DR): the risk of DR is almost twice as high among those who develop diabetes below 40 years of age.^4^ Assuming that sight-threatening DR (STDR) affects 10% of people living with diabetes, the number of people with STDR would increase to 10 or 11 million by 2030. Effective screening programmes embedded in existing health systems at all levels, coupled with treatment facilities nearby, should be strongly emphasised over the next two decades. Task-shifting will need to be considered as screening such a large number of diabetes will be beyond the reach of ophthalmologists in South Asia. Countries should develop national policies on training, deployment and utilisation of the different types of eye care professionals supporting the health system.

Age-related macular degeneration will also increase in the next two decades, and there will be an increasing need to develop low vision centres to support people who suffer from the condition.

With an increasing incidence of premature babies who survive, there is now a greater risk of retinopathy of prematurity (ROP) across South Asia, especially in urban areas. Integrating screening for ROP in care units for sick and premature babies, and in child care centres or programmes, is of immediate importance.

### Vision centres

Vision centres supported by tele-consultation or other forms of remote reporting must be developed as comprehensive eye care centres and not be limited to refractive services alone. Identifying refractive errors, and dispensing affordable corrective spectacles, should be a core function of vision centres, but not their only activity. There has to be regular monitoring of skills and outcomes in order to enhance the quality of the services offered at this level. Their work can be supported by non-communicable disease volunteers who can be trained to detect conditions and encourage compliance with treatment. Vision centres should be integrated or linked with the primary health care network so that resources can be optimised.

### Gender

The South Asian community has a strong male preference. This gender disparity also affects service uptake by women. Therefore, sex-disaggregated data should become a norm when monitoring performance. Innovative initiatives and incentives may be required in order to bring about change. This is critical, as female life expectancies outstrip that of men by a large margin, and widowed, dependent women will require greater support from the health systems.

### Training

With institutions of excellence in some of the countries in South Asia demonstrating their capacity to support eye care in other low- and middle-income countries, South-South collaboration needs to be augmented. This is not only cost-effective but there is better appreciation of the cultural needs of supported populations. Except for Nepal and Bhutan, all the other countries in South Asia were colonies of the United Kingdom and are therefore members of the Commonwealth. This platform can and should be used to strengthen transnational eye care services.

Inadequacies in training curricula for ophthalmology residents have been highlighted in the recent past. Deficiencies exist in exposure to diagnostic and surgical methods. Certification and validation procedures are rarely practiced in the region and clinical audits are not routinely conducted. These aspects need to be addressed to improve quality. With improved access to information technology platforms, the use of massive open online courses should be promoted within the region (see pp 93–95). Doing so not only reduces cost, but helps with the standardisation of service delivery norms, because people are exposed to best-practice models.

Health, including eye health, must be placed at the centre of the political debate and political agenda across South Asia. Even though families face catastrophic health expenditures, health is not a priority at the community level. This script needs to be changed by means of strong advocacy.

The issue today is not about technology. South Asia is the hub of global information technology and high quality service delivery protocols are available. The challenge confronting South Asia is how to use the available technology to achieve social transformation.
